# Generation of concentric perfect Poincaré beams

**DOI:** 10.1038/s41598-019-50705-z

**Published:** 2019-10-25

**Authors:** Zhongzheng Gu, Da Yin, Fengyan Gu, Yanran Zhang, Shouping Nie, Shaotong Feng, Jun Ma, Caojin Yuan

**Affiliations:** 1 Jiangsu Key Laboratory for Opto-Electronic Technology, School of Physics and Technology, Nanjing Normal University, Nanjing, 210023 China; 2 Jiangsu Center for Collaborative Innovation in Geographical Information Resource Development and Application, Nanjing, 210023 China; 3 School of Electronic Engineering and Optoelectronic Techniques, Nanjing University of Science and Technology, Nanjing, 210094 China

**Keywords:** Optical physics, Imaging and sensing, Liquid crystals

## Abstract

We theoretically propose and experimentally verify a method to generate new polycyclic beams, namely concentric perfect Poincaré beams (CPPBs), by using an encoded annular phase mask. The proposed beams consisting of multiple polarization structured fields can be simultaneously generated in one concentric mode, which are respectively mapped by fundamental Poincaré sphere (PS), high-order Poincaré sphere (HOPS), and hybrid-order Poincaré sphere (HyPS). Moreover, the ring radius, numbers and polarization orders of the CPPBs at arbitrary positions on arbitrary PS are independently controlled. This work enriches the mode distributions of perfect vortex and introduces a new polarization degree of freedom, which has the potential to implement more information beyond the orbital angular momentum multiplexing in optical communication.

## Introduction

Optical vortex beams carrying orbital angular momentum have attracted considerable attention in many exciting fields such as optical communication^1–5^, optical trapping^6,7^, optical imaging^8^, optical measurement^9,10^, quantum information processing^11–13^ and so on. For optical communication, OAM provides a new degree of freedom for multiplexing to achieve capacity beyond conventional multiplexing techniques. Since the ring radius of vortex intensity profile strongly depends on the topological charge, it is difficult to couple multiple OAM beams into a single fiber used for multiplexed communications simultaneously^14,15^. To make the diameter irrespective of topological charge, Ostrovsky *et al*. proposed the concept of the perfect vortex beam which has the same diameter with different topological charges of the spiral phase^16,17^. Furthermore, Panchanratnam-Berry phase method^18^ and combined modulation method of geometric and dynamic phase^19^ were proposed to flexibly generate perfect vector beams. Taking advantage of this property, Chen *et al*. successfully utilized the perfect vortex beam to perform particle manipulation^20^, following which its application has extended to the optical fiber communication field^21^.

However, the advantage of perfect vortex beam also is the root of its predicament, which makes it unable to meet the various applications. To overcome the limitations of perfect vortex beam, Li *et al*. realized circular optical vortex array^22^ and close-packed optical vortex lattices^23^. In 2016, Zhao *et al*. expanded the concept of perfect vortex to perfect vector vortex domain. Recently, Zhang *et al*. proposed the concept of concentric perfect vortex beams by using an amplitude hologram^24^. However, the degree of freedom of polarization, as an important parameter in OAM multiplexing technology, cannot be freely controlled, which limits the multiplexing capacity in optical communication. To visually present the states of polarization and phase, the concentric beams which are of isotropic and anisotropic polarizations can be represented by a prominent geometry such as a fundamental PS^25^, a HOPS^26,27^ and a HyPS^28^, where the state of polarization can be described as a point on the surface of a unit sphere. States on fundamental PS, HOPS, and HyPS are uniformly referred to Poincaré beams, which describe almost all polarization structured fields^29^. Therefore, it is of great interest to design novel form of phase patterns and experimental setup for generating three categories of Poincaré beams simultaneously, which contains fundamental PS, HOPS, and HyPS.

In this letter, we successfully generate CPPBs with the same center, controllable polarization orders and arbitrarily tuned ring radius and numbers, where the CPPBs can be at arbitrary positions on the surface of arbitrary order PS. Due to the richness of polarization and OAM multiplexing of CPPBs, this method of generating CPPBs has the potential to greatly increase communication capacity.

## Results

### Theoretical analysis

In the paraxial approximation, a generalized Poincaré beam on the surface of arbitrary order PS can be represented as^26,28^.

1 $$|\psi \rangle ={\psi }_{R}^{m}|{R}_{m}\rangle +{\psi }_{L}^{n}|{L}_{n}\rangle $$

and

2 $$\begin{array}{ccc}|{R}_{m}\rangle  & = & exp(im\varphi \mathrm{)[1,}i{]}^{T}/\sqrt{2}\\ |{L}_{n}\rangle  & = & exp(in\varphi \mathrm{)[1,}-\,i{]}^{T}/\sqrt{2}\end{array}$$

where $|{R}_{m}\rangle$ and $|{L}_{n}\rangle$ represent the right and left circularly polarized vortex beams with topological charges of *m* and *n*, respectively. The polarization state on the arbitrary order PS is mapped by representing the Stokes parameters *S*_0_, *S*_1_, *S*_2_, and *S*_3_ in the spherical Cartesian coordinates. These parameters are defined as^30^.

3 $$\{\begin{array}{ccc}{S}_{0} & = & |{\psi }_{R}^{m}{|}^{2}+|{\psi }_{L}^{n}{|}^{2}\\ {S}_{1} & = & \mathrm{2|}{\psi }_{R}^{m}||{\psi }_{L}^{n}|cos\phi \\ {S}_{2} & = & \mathrm{2|}{\psi }_{R}^{m}||{\psi }_{L}^{n}|sin\phi \\ {S}_{3} & = & |{\psi }_{R}^{m}{|}^{2}-|{\psi }_{L}^{n}{|}^{2}\end{array}$$

where $\phi =arg({\psi }_{R}^{m})-arg({\psi }_{L}^{n})$, and $|{\psi }_{R}^{m}{|}^{2}$ and $|{\psi }_{L}^{n}{|}^{2}$ are the intensities of $|{R}_{m}\rangle$ and $|{L}_{n}\rangle$ eigenstates, respectively.

**Figure 1:**
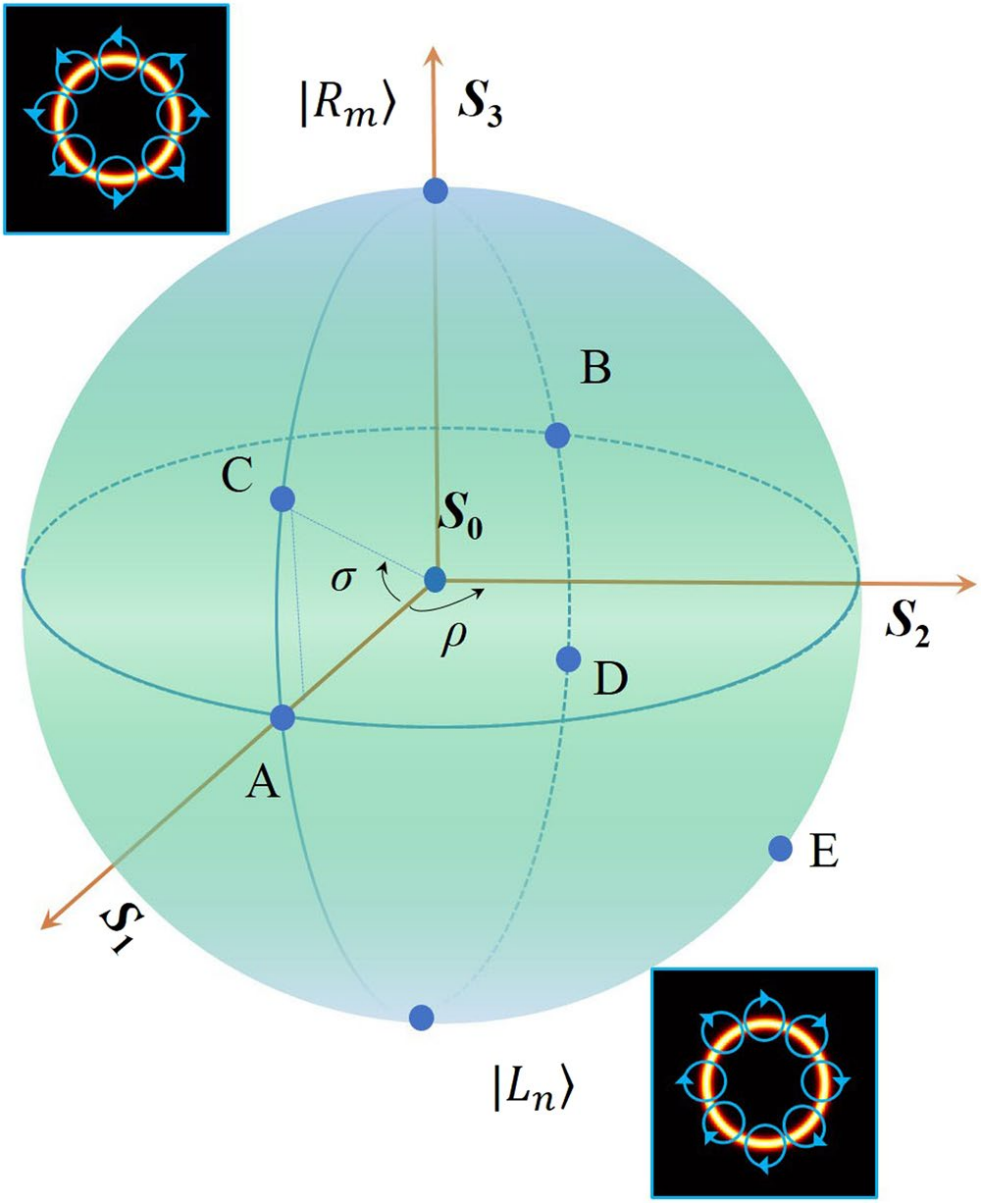
Schematic illustration of the arbitrary order PS. The north pole and south pole represent the right and left circularly polarized vortex beams with topological charges of *m* and *n*, respectively. Point A(0, 0), B(*π*, 0), C(0, *π*/6), D(*π*, −*π*/6) and E(*π*/2, −*π*/4) represent different states of polarization on the surface of arbitrary order PS.

As shown in Fig. 1, an arbitrary order PS can be denoted by *S*_0_, *S*_1_, *S*_2_, and *S*_3_ on a unit sphere. The north pole and south pole represent orthogonal circularly polarized eigenstates, i.e., the eigenstates $|{R}_{m}\rangle$ and $|{L}_{n}\rangle$. The longitude *ρ* and the latitude *σ* indicating the position of the polarization state on arbitrary order PS can be written as

4 $$\{\begin{array}{rcl}\rho  & = & arctan({S}_{2}/{S}_{1})=\phi \\ \sigma  & = & arcsin({S}_{3}/\sqrt{{S}_{1}^{2}+{S}_{2}^{2}+{S}_{3}^{2}})=arcsin({S}_{3}/{S}_{0})\end{array}$$

**Figure 2:**
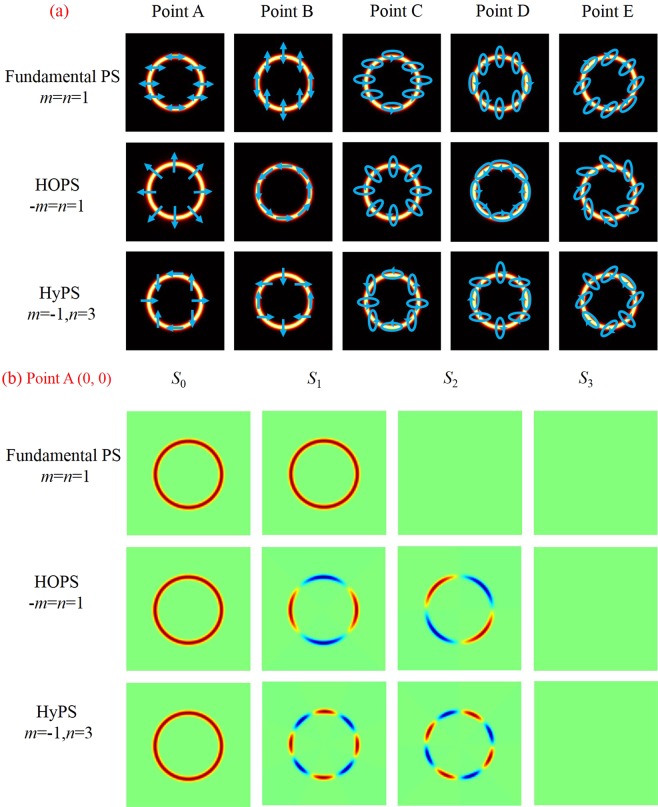
(**a**) The states of polarization of Point A (0, 0), B (*π*, 0), C (0, *π*/6), D (*π*, −*π*/6) and E(*π*/2, −*π*/4) on the surface of fundamental PS (*m* = *n* = 1), HOPS (−*m* = *n* = 1), and HyPS (*m* = −1, *n* = 3). (**b**) Stokes parameters *S*_0_, *S*_1_, *S*_2_, and *S*_3_ of different Poincaré spheres at point A.

According to the above formula, the longitude *ρ* represents the phase difference *ϕ* between the orthogonal right and left circularly polarized eigenstates. It is clear that an arbitrary perfect Poincaré beam (PPB) can be denoted on this sphere and characterized by the polarization order *P* = (*n* − *m*)/2 and the topological Pancharatnam charge *l*_*p*_ = (*n* + *m*)/2^31^. Particularly, when *m* = n, the PS becomes the fundamental PS. If −*m* = n, the PS evolves into HOPS. It becomes HyPS which has a more general form when |*m*|≠|*n*|. Therefore, on the surface of different Poincaré spheres, the points A(0, 0), B(*π*, 0), C(0, *π*/6), D(*π*, −*π*/6) and E(*π*/2, −*π*/4) represent distinct polarization states. For instance, the intensity distributions of points A, B, C, D and E on the sphere represented by fundamental PS (*m*_1_ = *n*_1_ = 1), HOPS (−*m*_2_ = *n*_2_ = 1), and HyPS (*m*_3_ = −1, *n*_3_ = 3) are shown in Fig. 2(a). It is obvious that the same point has different polarization states on different Poincaré spheres. As shown in the first column of Fig. 2(a), the point A(0, 0) represents horizontal linear polarization when it is a fundamental PS; On the surface of HOPS, the point A corresponds to radial linear polarization; When it evolves into HyPS, the point A has a more general polarization distribution. Particularly, the linear polarizations of the PPBs are always located on the equator. In Fig. 2(b), the point A is selected to show different theoretical stokes parameters^32,33^ with respect to different PS.

**Figure 3:**
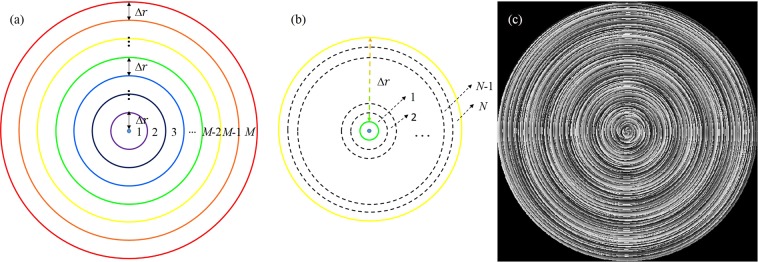
Schematic diagram of the encoded annular phase mask.

In order to generate the CPPBs, an encoded annular phase mask is produced using the following equation

5 $$\{\begin{array}{ccc}{H}_{1} & = & \mathop{\sum }\limits_{j=1}^{MN}\{{P}_{j}\cdot exp[i({m}_{\gamma }\theta +2\pi r/{d}_{\gamma }+{\phi }_{\gamma })]\}\\ {H}_{2} & = & \mathop{\sum }\limits_{j=1}^{MN}\{{P}_{j}\cdot exp[i({n}_{\gamma }\theta +2\pi r/{d}_{\gamma }+{\phi }_{\gamma }^{\text{'}})]\}\end{array}$$

and

6 $$\begin{array}{ccc}{P}_{j} & = & \{\begin{array}{cc}1, & \frac{(j-\mathrm{1)}{r}_{0}}{MN}\le r\le \frac{j{r}_{0}}{MN}\\ 0, & other\end{array}\\ \gamma  & = & j-N\cdot Round[(j-N)/N]\end{array}$$

where *H*_1_ and *H*_2_ are the two phase patterns loaded on each half of the SLM. The parameters *m* and *n* represent the topological charge of $|{R}_{m}\rangle$ and $|{L}_{n}\rangle$, respectively. The coordinate (*r*, *θ*) denotes the polar coordinate at the object plane. *φ*_*γ*_ and ${{\phi }^{{\rm{^{\prime} }}}}_{\gamma }$ are the initial phase and *d*_*γ*_ is the radial period of the axicons. The *P*_j_ item represents annular apertures which have the same ring width, *r*_0_ is the maximum radius of the encoded annular phase mask limited by the size of SLM. *Round*[] denotes a rounding function and *γ* is an integer ranging from 1 to *N*. As shown in Fig. 3(a,b), the encoded annular phase mask is divided into *M* parts, and each annulus is then divided into *N* smaller annulus^34^. Besides, *N* is used to control the number of perfect vortex beams and *M* is used to control the uniformity of the generated beams. The spiral axicons add annular apertures, and consequently the encoded annular phase mask is produced. For instance, the encoded annular phase mask for *M* = 20, *N* = 3 (*m*_1_ = 1, *m*_2_ = 4, *m*_3_ = 3) is illustrated in Fig. 3(c).

## Experiment and Results

**Figure 4:**
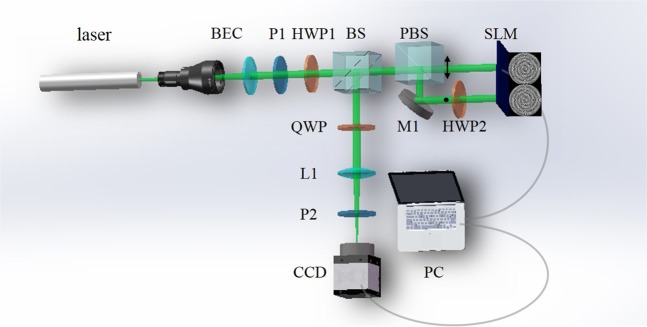
Experiment setup of the proposed CPPBs system.

A schematic of the experimental setup is shown in Fig. 4. A single-mode solid-state laser is used as the light source. After being expanded and collimated (BEC), the laser beam is transformed into a linearly polarized plane beam. Its polarized direction is determined by a polarizer (P1) and a half-wave plate (HWP1). Then, this linearly polarized beam is split into horizontally and vertically polarized beams by a polarizing beam splitter (PBS). Note that the ratio of horizontally and vertically polarized beams can be adjusted by rotating the HWP1. The horizontally polarized beam is reflected by a mirror M1, the propagating direction of which is parallel to the vertical one. The horizontally polarized beam illuminates the left half while the vertically polarized beam illuminates the right half of the reflective liquid-crystal spatial light modulator (SLM) screen, where two combined annular phase masks are addressed. Since the SLM can only modulate the horizontally polarized component of incident beam, a HWP2 is placed between the mirror M1 and SLM to transform vertically polarized beam into horizontally polarized beam before being reflected. After that, HWP2 changes the polarization direction again. The horizontally and vertically polarized beams are recombined and superposed by the PBS. Then the two components are converted to orthogonal circular polarizations via a quarter-wave plate (QWP). Finally, with a convex lens (L1), the concentric PPBs are recorded by using a charge-coupled device (CCD) camera at the focal plane.

**Figure 5:**
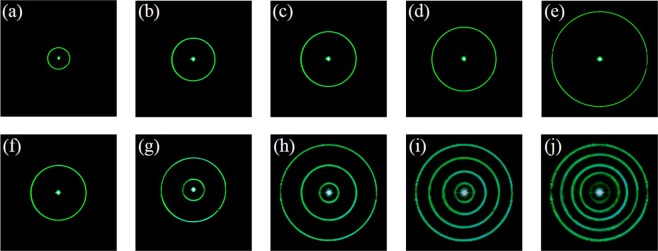
From left to right, the generated PPBs with different ring radius are shown in the upper row. In the bottom row, different numbers of ring are shown.

Theoretically, countable CPPBs with independent ring radius are obtained arbitrarily. The axicon parameter *d*_*γ*_ controls the ring radius. In Fig. 5(a–e), it is shown that ring radius of PPBs increase when the axicon parameter *d*_*γ*_ decreases. Figure 5(f–j) contains 1, 2, 3, 4, and 5 CPPBs which have the same center and different ring radius, respectively. The topological charges of the two orthogonal components have the same values *m* = n = 1, therefore the polarization order is set as *P* = 0 and topological Pancharatnam charge *l*_*p*_ = 1. However, because of the inherent defect of SLM, the diffracted beam has a bright spot in the optical field center.

**Figure 6:**
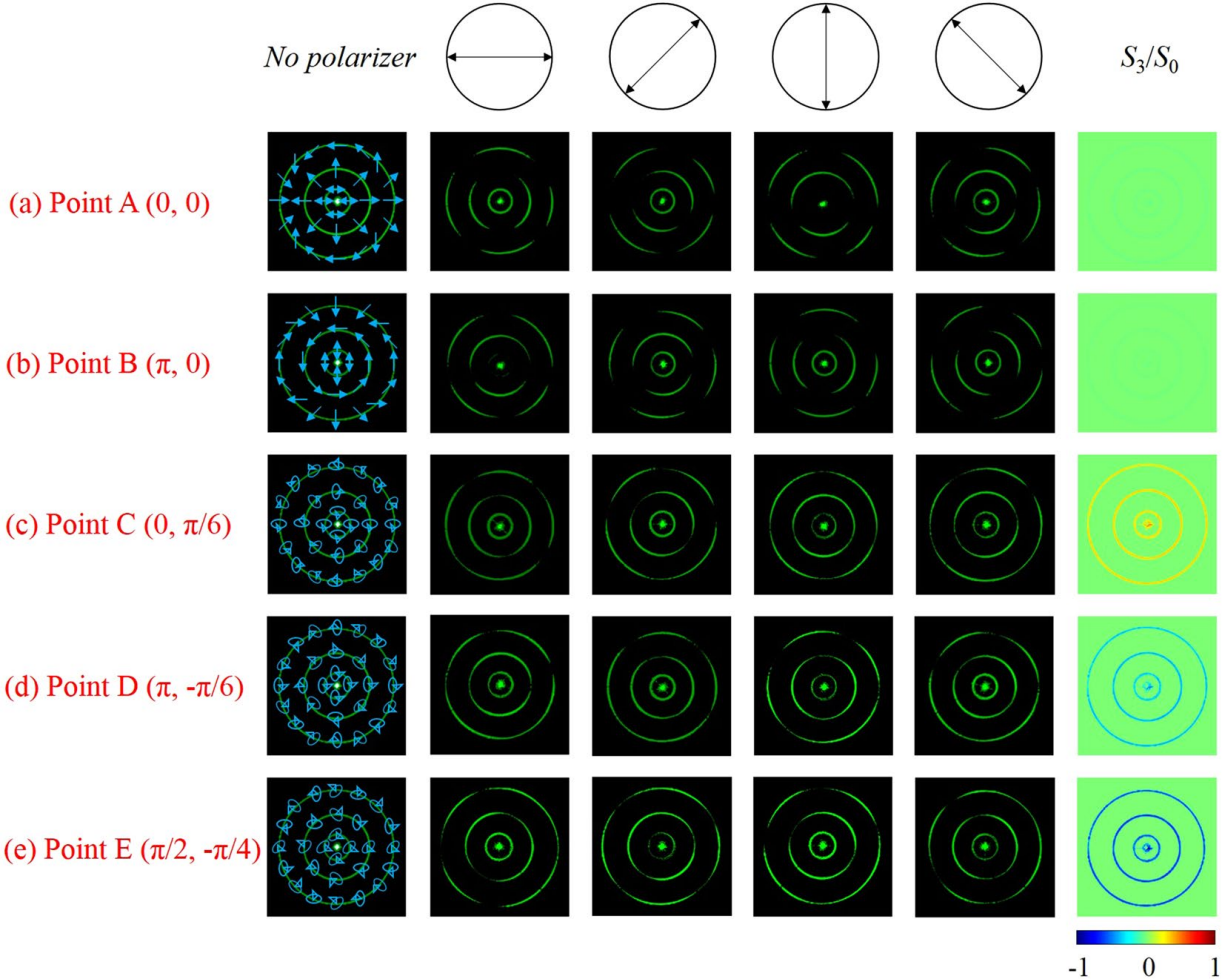
The CPPBs at different points on the surface of arbitrary order PS are generated when *m*_1_ = *n*_1_ = 1, −*m*_2_ = *n*_2_ = 1, *m*_3_ = −1, *n*_3_ = 3. Point A, B, C, D and E are illustrated in (**a**–**e**), respectively. From left to right are the recorded patterns by CCD with a rotating polarizer P2. The corresponding experimental normalized Stokes parameters of CPPBs are shown in the last column.

According to Eq. 4, when the points like A (0, 0) and B (*π*, 0) are on the equator, the ratio *S*_3_/*S*_0_ should be 0 and the latitude *σ* is 0°. When the points at (0, *π*/6), (*π*, −*π*/6), (*π*/2, −*π*/4), the north and south pole, the ratio *S*_3_/*S*_0_ is 0.5, −0.5, −0.707, 1 and −1, and the latitude *σ* is 30°, −30°, −45°, 90°, and −90°. As HWP1 is rotated, the ratio of two components changes, resulting in the variety of the parameter *σ*. Besides, the parameter *ρ* varies with the phase difference between *φ*_*γ*_ and ${\phi ^{\prime} }_{\gamma }$. Therefore, we can obtain arbitrary polarization state on the arbitrary order PS, which can be shown by the inner ring, the middle ring, and the outer ring, respectively. For instance, by changing the orientation of the HWP1, we can generate elliptical polarization or circular polarization at the north pole and south pole or linear polarization at point A and B on the meridian of the sphere. As shown in Fig. 6, the CPPBs at the points A, B, C, D and E are generated by setting corresponding initial phase and changing the ratio between the intensities of orthogonal eigenstates. When the initial phase difference is equal to zero, the generated CPPBs shown in Fig. 6(a,c) can be changed from point A to point C by rotating the HWP1. Similarly, in Fig. 6(b,d), the points B and D are obtained when the phase difference is equal to *π* by rotating the HWP1. It is proved that CPPBs at arbitrary position at the surface of arbitrary order PS can be generated in this experimental setup. Experimentally, the corresponding ratios of *S*_3_/*S*_0_ representing the latitudes *σ* are shown in the last column of Fig. 6.

**Figure 7:**
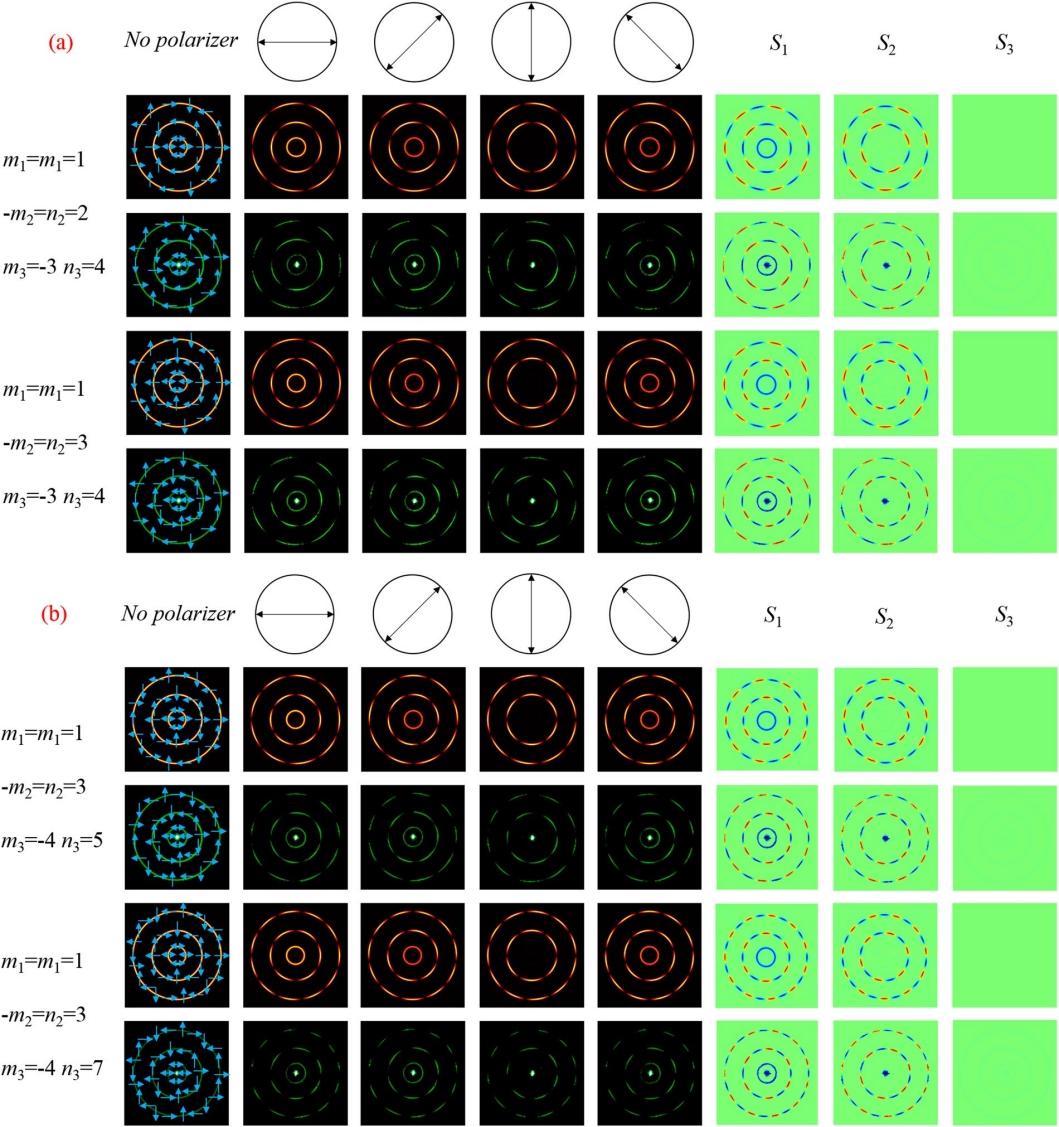
Three CPPBs with different topological charges. In (**a**), simulation and experimental results of the three CPPBs are shown when *m*_1_ = *n*_1_ = 1, *m*_3_ = −3, *n*_3_ = 4. In (**b**), simulation and experimental results of the three CPPBs are shown when *m*_1_ = *n*_1_ = 1, −*m*_2_ = *n*_2_ = 3. From left to right are the recorded patterns by CCD without or with a rotating polarizer. The Stokes parameters are shown in the last three columns.

In order to illustrate CPPBs better, the simulation and experimental results of CPPBs which have three concentric rings are shown in Fig. 7. The coordinates of these CPPBs is (0, 0), i.e. point A of Fig. 2. The inner ring represents fundamental PS, where the orthogonal components have identical topological charges (*m*_1_ = n_1_ = 1). Therefore, the scalar linear polarization in the equator of the fundamental PS is generated. Firstly, the topological charges of middle ring are changed when that of other two rings stay constant. As shown in Fig. 7(a), we set the topological charges of the inner ring and the outer ring as *m*_1_ = n_1_ = 1, *m*_3_ = −4, *n*_1_ = 7, respectively. Besides, we change the topological charge of the middle ring from −*m*_2_ = *n*_2_ = 2 to −*m*_2_ = n_2_ = 3. As a result, the polarization order *P* changes from 2 to 3 but the topological Pancharatnam charge of *l*_p_ keeps constant. In this condition, the distribution of polarization is also changed and a radially polarized beam can be acquired. Secondly, we keep the topological charges of the inner and middle ring unchanged and only adjust that of the outer ring. Therefore, the states of polarization represented by HyPS are transformed. Figure 7(b) respectively illustrates the situation of *m*_3_ = −4, *n*_3_ = 5 and *m*_3_ = −4, *n*_3_ = 7 when the topological charges of the inner and middle ring are set as *m*_1_ = n_1_ = 1 and −*m*_2_ = *n*_2_ = 2. As a result, the polarization order *P* alters from 4.5 to 5.5. It is noted that the inner ring has isotropic polarization but the remaining two rings have anisotropic polarization. The polarization distribution of CPPBs is analyzed by rotating the polarizer P2. Besides, in last three columns of Fig. 7, the theoretical and experimental Stokes parameters *S*_1_, *S*_2_ and *S*_3_ are measured to analyze the polarization distribution.

## Discussion

In summary, we have proposed the concept of CPPBs with respect to traditional concentric perfect vortex beams, and experimentally generated such beams. The experimental results are in good coincidence with the theory. Any desired CPPBs can be produced and analyzed through this flexible and simple setup. Due to the independently controllable topological charges, arbitrarily tuned ring radius and numbers, and the arbitrary state of polarization, the method is of great potential in fiber-optic multiplex communication.

## Methods

Figure 4 shows a schematic of the experimental setup. A 532 nm single-mode solid-state laser with 300 mw is used as the light source. A linearly polarized beam which has arbitrary linearly polarized direction can be obtained by a polarizer (P1) and a half-wave plate (HWP1). Then this linearly polarized beam is split into horizontally and vertically polarized components (i.e., *p*-component and *s*-component) by a polarizing beam splitter. It is noted that the ratio of *p*-component and *s*-component can be adjusted by rotating the half-wave plate because of the presence of PBS. According to Eq. 1, we can change the latitude *σ* by adjusting the ratio of two orthogonal eigenstates. The longitude *ρ* can be changed by setting the initial phase difference *ϕ* between the orthogonal eigenstates.The two components illuminate the left and the right halves of the reflective liquid-crystal spatial light modulator screen (Holoeye Pluto VIS, 1920 × 1080 pixels, pixel pitch is 8 *μ*m), respectively. Since the SLM can only modulate the horizontally polarized component of the incident beam, a HWP2 is placed between the mirror M1 and SLM to transform vertically polarized beam into horizontally polarized beam. Then the optical field of each component generated by the annular phase mask approximately presents *m* th or *n* th order Bessel-Gauss pattern. Finally, after passing through a quarter-wave plate, the CPPBs are generated and recorded by using a CCD (pixel pitch is 6.4 *μ*m) at the focal plane of L1 (*f* = 120 mm).
